# A cancer graph: a lung cancer property graph database in Neo4j

**DOI:** 10.1186/s13104-022-05912-9

**Published:** 2022-02-14

**Authors:** David Tuck

**Affiliations:** VA Boston Healthcare System, 150 South Huntington Avenue, Boston, MA 02130 USA

**Keywords:** Non-small cell lung cancer, Property graph database, The Cancer Genome Atlas

## Abstract

**Objectives:**

A novel graph data model of non-small cell lung cancer clinical and genomic data has been constructed with two aims: (1) provide a suitable model for facilitating graph analytics within the Neo4j framework or through tools which can interact through existing Neo4j APIs; and (2) provide a base model extensible to other cancer types and additional datasets such as those derived from electronic health records and other real world sources.

**Data description:**

Clinical and genomic data integrated with a novel property graph database schema from publicly available datasets and analyses based on The Cancer Genome Atlas lung cancer datasets augmented by with subgraphs patient-patient social network from similarity and correlation as well as individual based biological networks.

## Objective

The pathobiology of cancer involves the coordinated dysregulation of multiple processes across molecular, cellular, tissue, and organism scales [1]. Somatic mutations and genomic aberrations are crossed and intertwined with an individual patient’s clinical, social, and medical histories. The complex interrelationships among all of these factors determine disease origin, trajectory, and outcomes of interventions [2]. Strategies that allow operation directly on the topology of the graph structures defined by these relationships are enabled by the development and growing maturity of native graph databases such as TigerGraph and Neo4j [3–5].

This note describes a representation of non-small cell lung cancer in Neo4j, a property graph database platform which natively stores and processes graph data models. A version is available in a GPL3-licensed open-source community edition [6].

Non-small cell lung cancer is the most common cause of cancer deaths worldwide [7, 8], and it has plentiful publicly available genomic, clinical, and molecular data [9–11]. The lung cancer graph database provides an analytic framework for integrating modeling of disease mechanisms on a genome-scale and clinical data from clinical electronic health records, diagnostic studies, therapeutic interventions, and molecular assays. This project utilizes several publicly available open data sources and extends these with calculated variables defining relationships to create a novel graph schema and nested set of subgraphs comprising the Neo4j database. Clinical, demographic, diagnostic, therapeutic, and multiple genomic measures are obtained from TCGA LUAD and LUSC datasets [9, 10]. Multiple analyses have extended available attributes for immunologic, and biologic signaling pathway profiles [11–15] enabling the creation of a graph structure at different scales based on relations among cancer cases, relations among biological molecules, relations of biological networks and processes within individual patients.

By adopting graph database technology, this data resource aims to provide a platform to explore the utility of integrative graph-based systems biology analyses to decode the molecular and clinical underpinnings of complex diseases.

## Data description

All data files and datasets are deposited in the Harvard Dataverse repository in dataset “A Cancer Graph: A Lung Cancer property graph using Neo4j” [16]. A file containing the entire graph database is provided as a binary database dump (Data file 1 in Table 1). The schema for the property graph is described in Dataset 2 which contains a graphic image of the schema and a json file containing the schema with all entities, attributes, and relationships among all the different entities. Data file 3 contains the commands for generating documentation of the schema, and indexes, for loading the binary file into a Neo4j instance. Data file 3 also provides example commands in the cypher language used by Neo4j which describes how the database was originally generated from input files. These individual input data source files are provided (Dataset 4 in Table 1) as comma separated value formatted files.

**Table 1:** Overview of data files/data sets

Label	Name of data file/data set	File types (file extension)	Data repository and identifier (DOI or accession number)
Data file 1	lung-cancer-graph-neo4j-2021-07-15T022024.bin	binary dump file (bin)	Harvard Dataverse [16] https://doi.org/10.7910/DVN/RIXLG8
Data file 2	README.md	text/markdown	Harvard Dataverse [16] https://doi.org/10.7910/DVN/RIXLG8
Data file 3	ACancerGraphSchema.png	image (png)	Harvard Dataverse [16] https://doi.org/10.7910/DVN/RIXLG8
Data file 4	makeIndexesConstraints.cql	cypher (cql)	Harvard Dataverse [16] https://doi.org/10.7910/DVN/RIXLG8
Data file 5	ACancerGraphLoader.cql	cypher (cql)	Harvard Dataverse [16] https://doi.org/10.7910/DVN/RIXLG8
Data file 6	schema.json	json	Harvard Dataverse [16] https://doi.org/10.7910/DVN/RIXLG8
Data set 1	Input files (csv format) to create database	comma separated values (csv)	Harvard Dataverse [16] https://doi.org/10.7910/DVN/RIXLG8
Data file set 2	Input files (csv format) to create supportive data	comma separated values (csv)	Harvard Dataverse [16] https://doi.org/10.7910/DVN/RIXLG8

The property graph database consists of (a) publicly available open access data of patients with non-small cell lung cancer and (b) derived variables augmented by relationships defining different subgraphs. The database contains data from > 1000 patients from the Cancer Genome Atlas (TCGA) which contain clinical, diagnostic, and therapeutic data (chemotherapy, radiation, immunotherapy), as well as multiple genomic measures (gene expression, somatic, mutations, copy number, epigenetics). Additional attributes are derived from independent published analyses based on these data, providing signatures related to immunologic, DNA repair, molecular portrait subtypes, and profiles from a variety of biological pathways [11–15]. The dataset also incorporates relevant portions of precedent native graph representations of biological and biomedical systems including Hetio [17, 18] and Reactome [19], both of which use Neo4j platform to represent complex biological networks. This existing framework is supplemented by pathway, genomic and various calculated variables including graph kernels, embedded vector representation of somatic gene mutations, and computed pathway activations.

The primary value of the dataset come from calculated relationships which create subgraphs that serve as a substrate for the application of exploration and application of graph algorithms [20–22]. These occur primarily at two different scales: (1) patient-patient network with direct relationships among patients (or tumor samples) based on similarity scores or correlation for genomic features or signatures; (2) biological networks within single patient samples.

- CancerCase (Patient-based) networks provide graphs of the relationships between patients based on calculation of similarity and correlation scores of molecular signatures such as immune scores or DNA repair profiles.
- Intra-patient biological signaling activation networks InFlo [14] is a robust systems biology approach for integrative analysis of multi-omics data which can characterize complex biological signaling network activities in any given biological sample. InFlo was applied for individual samples from TCGA including the non-small cell lung cancer samples/Thus calculating a complete biological network activation state for each individual tumor sample.

In summary, a novel graph data model has been constructed integrating clinical and molecular data of non-small cell lung cancer patients with aims: (1) a graph model for facilitating graph analytics within the Neo4j framework or through tools via the Neo4j application programming interface (API); and (2) exploratory basis extension to other tumor types or clinical datasets derived from electronic health records.

## Limitations

- The database is limited in the number of variables, which may not satisfy specific needs.
- The schema of the database.
- TCGA is rich in omics but relatively poor in clinical details (comorbidity, frailty assessment, specific lab results, extended pharmacy).

And other sources with additional modifications (TCGA is rich in omics but relatively poor in clinical details (comorbidity, frailty assessment, specific lab results, extended pharmacy, etc.).

## Data Availability

The data described in this Data note can be freely and openly accessed at Harvard Dataverse: https://doi.org/10.7910/DVN/ZU [16]. Please see Table 1 and references [16] for details. Original data sets are available in the Cancer Genome Atlas repository, https://tcga-data.nci.nih.gov/tcga/. Inflo was developed in the Varadan lab at Case Western : https://varadanlab.github.io/InFlo/.

## Abbreviations

TCGA: The Cancer Genome Atlas
LUAD: Lung adenocarcinoma
LUSC: Lung squamous cell carcinoma
API: Application Programming Interface
